# Associations between caregiver-detected delirium and symptoms of depression and anxiety in family caregivers of critically ill patients: a cross-sectional study

**DOI:** 10.1186/s12888-021-03200-7

**Published:** 2021-04-09

**Authors:** Brianna K. Rosgen, Karla D. Krewulak, Judy E. Davidson, E. Wesley Ely, Henry T. Stelfox, Kirsten M. Fiest

**Affiliations:** 1 Departments of Critical Care Medicine and Community Health Sciences, Cumming School of Medicine, University of Calgary, Calgary, AB T2N 1N4 Canada; 2 Department of Critical Care Medicine, Cumming School of Medicine, University of Calgary, Calgary, AB T2N 1N4 Canada; 3 Department of Education, Development and Research, University of California, San Diego Health, San Diego, CA 92103 USA; 4 Tennessee Valley Veteran’s Affairs Geriatric Research Education Clinical Center (VA GRECC), Department of Medicine, Center for Health Services Research and Division of Pulmonary and Critical Care Medicine, Vanderbilt University Medical Center, Nashville, TN 37212-2637 USA; 5 Department of Critical Care Medicine, Department of Community Health Sciences, and O’Brien Institute for Public Health, Cumming School of Medicine, University of Calgary, Calgary, AB T2N 1N4 Canada; 6 Department of Critical Care Medicine, Department of Community Health Sciences, Department of Psychiatry, and O’Brien Institute for Public Health, Cumming School of Medicine, University of Calgary, Calgary, AB T2N 1N4 Canada; 7 Department of Critical Care Medicine, Ground Floor, McCaig Tower, 3134 Hospital Drive NW, Calgary, Alberta T2N 5A1 Canada

**Keywords:** Family, Caregiver, Intensive care, Depression, Anxiety

## Abstract

**Background:**

Witnessing delirium can be distressing for family caregivers (i.e., relatives or friends) of critically ill patients. This study aimed to evaluate associations between caregiver-detected delirium in critically ill patients and depression and anxiety symptoms in their family caregivers.

**Methods:**

Consecutive adult patient-caregiver dyads were enrolled from a 28-bed medical-surgical intensive care unit. Patient delirium was screened for daily by family caregivers using the Sour Seven instrument. Family caregivers completed the Patient Health Questionnaire-9 (PHQ-9) and General Anxiety Disorder-7 (GAD-7) instruments daily to assess their own depression and anxiety symptoms. Response feature analysis was used to handle repeated measures. Descriptive statistics and regression analyses were completed.

**Results:**

One hundred forty-seven patient-caregiver dyads were enrolled. Clinically significant symptoms of depression and anxiety occurred in 27% and 35% of family caregivers, respectively. Caregiver-detected delirium occurred in 65% of patients, and was not associated with clinically significant caregiver depression (Odds Ratio [OR] 1.4, 95% Confidence Interval [95%CI] 0.6–3.1) or anxiety (OR 1.2, 95%CI 0.6–2.6) symptoms. When stratified by Sour Seven scores, scores 1–3 and 4–9 were associated with increased symptoms of anxiety (OR 3.1, 95%CI 1.3–7.0) and depression (OR 2.6, 95%CI 1.1–6.1) in family caregivers. Caregiver-detected delirium score was associated with severity of family caregiver anxiety symptoms (coefficient 0.2, 95%CI 0.1–0.4), but not depression symptoms (coefficient 0.2, 95%CI -0.0–0.3).

**Conclusions:**

Caregiver-detected patient delirium was associated with increased depression and anxiety symptoms in family caregivers of critically ill patients. Further randomized research is required to confirm these associations.

## Background

Family caregivers are important to the care of critically ill patients in the intensive care unit (ICU), commonly acting as surrogate decision makers and providing support to their loved ones [1]. An ICU stay can be distressing for family caregivers due to uncertainty regarding their loved ones’ condition, inability to speak with their loved one, and witnessing their loved one in a critically ill condition [2]. Family caregivers have a high burden of anxiety and depression during an ICU stay, with 40–80% experiencing symptoms of anxiety, and 16–90% experiencing symptoms of depression [3]. Variability in prevalence estimates may be attributable to differences in population, measurement tools used, and follow-up time points [3].

Family caregivers present at the bedside often witness delirium, an acute state of confusion that affects between 20 and 50% of ICU patients [4]. Delirium can lead to significant distress and anxiety for both patients and family members [5]. Family caregivers are often present at the bedside and familiar with a patient’s baseline mental status and thus well-positioned to detect delirium. For these reasons, family caregivers may be especially useful for identifying delirium, particularly when delirium presents with subtle symptoms such as lethargy and withdrawal [6]. Two tools for family caregivers to detect delirium have been validated and deemed feasible to employ in ICU populations: the Family Confusion Assessment Method (FAM-CAM) and Sour Seven [7]. In an ICU sample, the Sour Seven performed similar to provider-administered delirium measurement tools in identifying delirium, highlighting the potential utility for involving family caregivers in delirium detection [7]. Additionally, meaningful involvement of family caregivers in patient care has demonstrated improvements in satisfaction with care and reduced distress [8, 9]. However, it is unknown how family participation in delirium detection impacts distress and adverse psychological outcomes in family caregivers.

Though several studies have identified an association between delirium and family caregiver distress [10–16], to our knowledge no studies have evaluated the association between participating in delirium detection and adverse psychological symptoms, such as depression and anxiety, in family caregivers of critically ill patients. As such, this study aims to evaluate: i) associations between family caregiver-detected delirium and the presence of clinically significant depression and anxiety symptoms in family caregivers, ii) associations between family caregiver-detected delirium score (categorical) and the presence of clinically significant depression and anxiety symptoms in family caregivers, and iii) associations between family caregiver-detected delirium score (continuous) and the severity of clinically significant depression and anxiety symptoms in family caregivers.

## Methods

### Participants and procedures

This cross-sectional study was a planned sub-study of a larger published validation study registered on ClinicalTrials.gov (NCT03379129) [7], and reported according to The Strengthening the Reporting of Observational Studies in Epidemiology (STROBE) guidelines for reporting observational studies [17]. Eligible patient-family caregiver dyads admitted to a 28-bed medical-surgical ICU at Foothills Medical Centre (a large tertiary care academic hospital in Calgary, Canada) between December 2017 and March 2019 were enrolled. Patients' eligibility criteria are presented in Table 1. Family caregivers were defined as any person who was present during the patient’s ICU stay and was familiar with the patient’s baseline behavior and cognitive functioning. Family caregivers were considered eligible if they accompanied an eligible patient, and were ≥ 18 years old, able to give informed consent, and able to understand English.

**Table 1:** Participant eligibility criteria

Inclusion Criteria	
≥18 years old	
Family caregiver present	
Richmond Agitation-Sedation Scale Score ≥ − 3	
Able to provide informed consent	
Able to communicate with research staff	
Anticipated to remain in the ICU for at least a further 24 h	
No new primary neurologic injury	
Glasgow Coma Scale score > 9	

Family caregivers completed the Sour Seven questionnaire once daily to evaluate delirium in their loved ones. Family caregivers completed the Patient Health Questionnaire 9 (PHQ-9) [18] and Generalized Anxiety Disorder 7 (GAD-7) [19] once daily to assess their symptoms of depression and anxiety, respectively. All questionnaires were administered for a maximum of 5 days during the ICU stay.

### Measures

#### Caregiver-detected delirium measure

Family caregivers detected delirium using the Sour Seven questionnaire. The Sour Seven contains seven weighted questions, totaling a maximum score of 18. Items evaluate features of delirium, including altered awareness and attention, fluctuation, disordered thinking and behavior, impaired eating or drinking, and difficulties with mobility [20]. Using a cutoff score of 4 or greater to indicate delirium in critically ill adults, the Sour Seven has a sensitivity of 72.9% and specificity of 68.8% [7]. Sour Seven scores were classified categorically (grouped into scores 1–3, scores 4–9, and scores 10–18) and continuously (range 0–18), for Objectives ii and iii, respectively.

#### Family caregiver depression and anxiety measures

The PHQ-9 is a self-administered 9-item scale that assesses symptoms of depression within the previous 2 weeks. PHQ-9 items represent the Diagnostic and Statistical Manual of Mental Disorders 4th Edition (DSM-IV) criteria for depression, and are scored ranging from 0 (not at all) to 3 (almost every day) [21]. Scores of 5, 10, 15, and 20 (maximum possible score of 27) represent cutoffs for mild, moderate, moderately severe and severe depression, respectively. Using a cutoff score of 10 or greater to indicate clinically significant depression, the PHQ-9 has a sensitivity and specificity of 88% [18].

The GAD-7 is a self-administered 7-item scale to assess symptoms of anxiety in the previous 2 weeks. The GAD-7 items represent the DSM-IV criteria for generalized anxiety disorder, and are scored ranging from 0 (not at all) to 3 (almost every day). Scores of 5, 10, and 15 (maximum possible score of 21) represent cutoffs for mild, moderate, and severe anxiety, respectively. Using a cutoff score of 10 or greater to indicate clinically significant anxiety, the GAD-7 has a sensitivity of 89% and specificity of 82% [19].

#### Covariate measures

Family caregivers completed a self-report questionnaire on the first day of enrolment to collect demographic variables, including age, sex, gender, and education status. Patient variables were extracted from eCritical, a bedside clinical information system validated for research purposes [22]. Patient variables extracted included age, sex, Acute Physiology and Chronic Health Evaluation II [APACHE-II] score, and admission category (i.e., medical, surgical, neurological, trauma).

### Statistical analysis

All statistical analyses were completed in Stata (StataCorp, College Station, Texas, USA). Descriptive characteristics of patients and family caregivers were quantified using summary measures (i.e., mean and proportion) and accompanying interval estimates. The two-sided alpha value used for all analyses was 0.05. Listwise deletion was used to address missing data, although all dyads had at least one complete set of delirium and family caregiver questionnaires. Response feature analysis was used for multiple observations per patient-family caregiver dyad, whereby the highest score for each measure was used in the analysis [23].

We estimated the prevalence of delirium and clinically significant depression and anxiety symptoms with accompanying 95% confidence intervals (95%CI). Multivariable logistic regression models were used to evaluate associations of caregiver-detected delirium presence (present/absent) and score (categorical: Sour Seven scores 1–3, Sour Seven scores 4–9, and Sour Seven scores 10–18) with the presence (present/absent) of clinically significant depression and anxiety symptoms in family caregivers. Multivariable linear regression models were used to evaluate the association between Sour Seven score (continuous) and severity of depression or anxiety symptoms (continuous). We evaluated effect modification and confounding using covariates identified a priori, including patient variables (age, sex, APACHE-II score, reason for admission, analgosedative medication use) and caregiver variables (age, sex, education status).

We conducted a subgroup analysis to estimate associations between individual caregiver-detected delirium symptoms and clinically significant depression and anxiety symptoms in family caregivers. We used multivariable logistic regression models with individual Sour Seven items as the exposure (present/absent), and clinically significant depression and anxiety symptoms as the outcome (present/absent). We evaluated effect modification and confounding using covariates identified a priori, including patient variables (age, sex, APACHE-II score, reason for admission, analgosedative medication use) and family caregiver variables (age, sex, education status).

### Sample size

Detailed justification for sample size is described elsewhere [7]. Briefly, the minimum number of participants required to achieve the primary objective of the larger study (i.e., to assess the validity of the Sour Seven questionnaire), given the prevalence of delirium in the study ICU was 147 patient-caregiver dyads. All dyads recruited for the larger study were included in the current sub-study.

## Results

### Sample characteristics

**Fig. 1:**
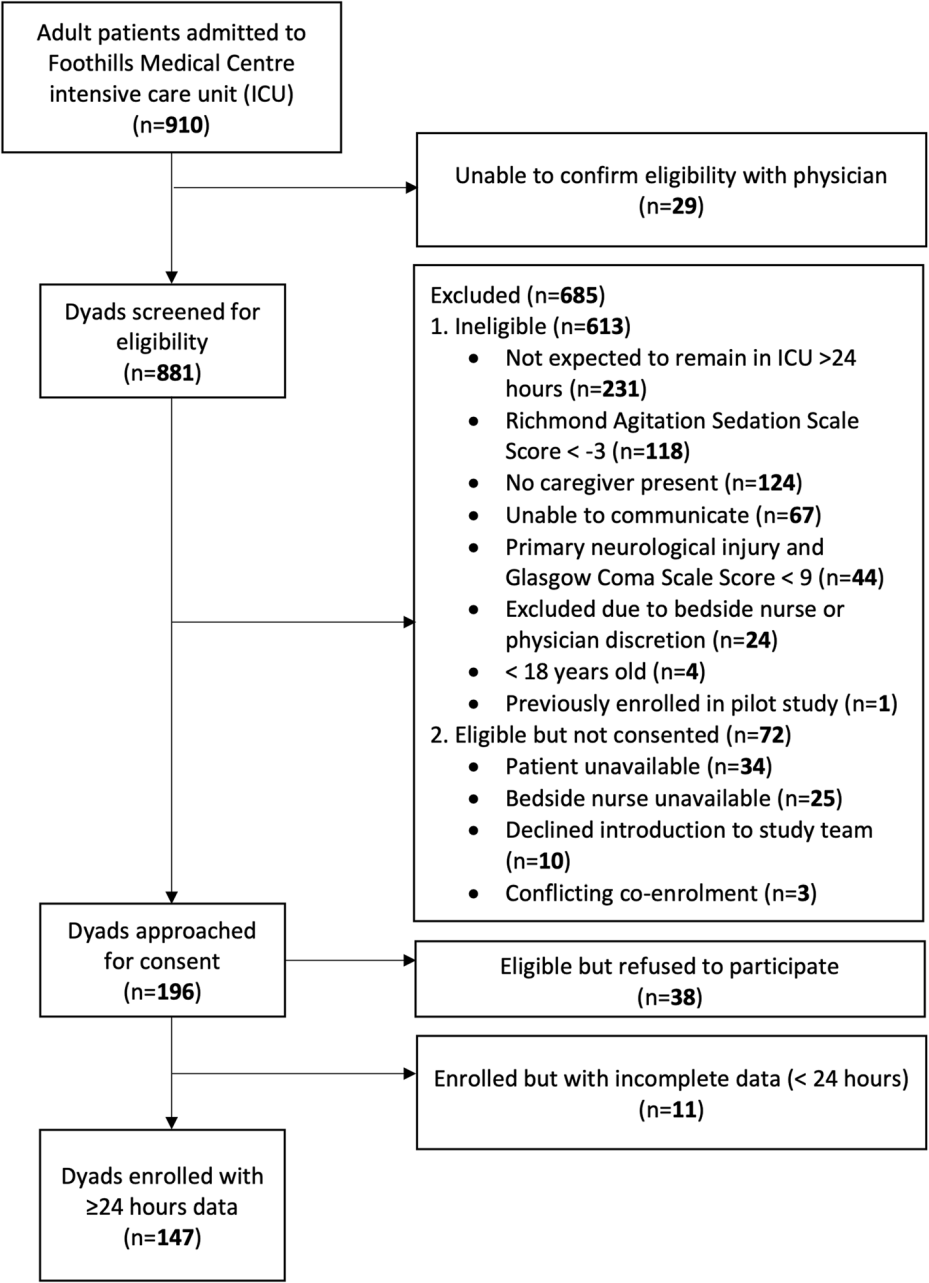
The Strengthening the Reporting of Observational Studies in Epidemiology (STROBE) participant flow diagram

Between December 2017 and March 2019, 910 patients were admitted to the study ICU, 196 were eligible and approached for consent, and 147 patient-caregiver dyads participated (Fig. 1). Patient and caregiver characteristics are presented in Table 2. The majority of admissions were classified as medical (45.6%, *n* = 67), followed by neurological (21.1%, *n* = 31), trauma (18.4%, *n* = 27), and surgical (15.0%, *n* = 22). The median (interquartile range, IQR) APACHE-II score was 20 (14–26). Most family caregivers were female (73.5%, *n* = 108), had a spousal relationship with the patient (48.3%, *n* = 71), and had a mean (±SD) age of 54.3 years (±14.3).

**Table 2:** Demographic characteristics of included patients and caregivers

Variable	Patients (*n* = 147)	Caregivers (*n* = 147)
Age, years, mean (±SD)	56.1 (16.2)	54.3 (14.3)
Sex, female, n (%)	58 (39.5)	108 (73.5)
Gender, woman, n (%)	58 (39.5)	108 (73.5)
Ethnicity or race, n (%)		
Black	2 (4.9)	
Caucasian	19 (13.4)	
East Asian	3 (2.1)	
Eastern European	6 (4.2)	
First Nations	2 (1.4)	
Hispanic	1 (0.7)	
Metis	1 (0.7)	
Middle Eastern	2 (1.4)	
North American	45 (31.0)	
Northern European	4 (2.8)	
South Asian	4 (2.8)	
South-East Asian	10 (7.0)	
Western European	41 (26.8)	
APACHE-II score, median (IQR)	20 (12)	–
ICU length of stay, median (IQR)	9.4 (9.4)	–
Reason for admission, n (%)		
Medical	67 (45.6)	–
Neurological	31 (21.1)	–
Trauma	27 (18.4)	–
Surgery	22 (15.0)	–
Level of education, n (%)		
High school or less	72 (49.7)	54 (37.0)
Some university/college education or greater	73 (50.3)	92 (63.0)
Relationship to patient, n (%)		
Spouse/common law	–	71 (48.3)
Child	–	34 (23.1)
Sibling	–	26 (17.7)
Other	–	3 (2.0)

*Abbreviations*: *SD* standard deviation, *IQR* 95% Interquartile range, *APACHE-II* Acute Physiology and Chronic Health Evaluation II

Approximately 65% (95%CI 56.5–72.0%) of patients had at least one positive Sour Seven score during their ICU stay, as detected by their family caregivers using the Sour Seven. In our sample, 26.5% (95%CI 20.0–34.3%) of family caregivers reported clinically significant symptoms of depression, and 35.4% (95%CI 28.0–43.5%) reported clinically significant symptoms of anxiety.

i) **Association between caregiver-detected delirium and clinically significant depression and anxiety symptoms in family caregivers**

There was no statistically significant association between caregiver-detected delirium in patients and clinically significant depression symptoms (Adjusted Odds Ratio, aOR 1.4, 95%CI 0.6–3.1), or clinically significant anxiety symptoms amongst family caregivers (aOR 1.2, 95%CI 0.6–2.6).

ii) **Association between caregiver-detected delirium score and clinically significant depression and anxiety symptoms in family caregivers**

Compared to patients who did not have delirium detected by their family caregivers, the adjusted OR of clinically significant depression symptoms for family caregivers who detected Sour Seven scores 1–3 was 2.0 (95%CI 0.8–5.0), Sour Seven scores 4–9 was 2.6 (95%CI 1.1–6.1) and Sour Seven scores 10–18 was 1.8 (95%CI 0.8–4.4) (Table 2). The adjusted OR of clinically significant anxiety symptoms for family caregivers who detected Sour Seven scores 1–3 was 3.1 (95%CI 1.3–7.0), Sour Seven scores 4–9 was 1.6 (95%CI 0.7–3.6), and Sour Seven scores 10–18 was 1.6 (95%CI 0.7–3.6) (Table 3).

iii) **Association between caregiver-detected delirium score and severity of depression and anxiety symptoms in family caregivers**

**Table 3:** Summary of associations between caregiver-detected delirium score category, measured using the Sour Seven, and presence of clinically significant depression and anxiety symptoms

Sour Seven score	Family caregiver depression presence		Family caregiver anxiety presence	
	Crude OR (95%CI)	Adjusted OR (95%CI)	Crude OR (95%CI)	Adjusted OR (95%CI)
1–3^a^	1.7 (0.7–3.9)	2.0 (0.8–5.0)	**2.5 (1.2–5.3)**	**3.1 (1.3–7.0)**
4–9^a^	**2.7 (1.2–5.9)**	**2.6 (1.1–6.1)**	1.5 (0.7–3.0)	1.6 (0.7–3.6)
10–18^a^	1.7 (0.8–3.9)	1.8 (0.8–4.4)	1.3 (0.6–2.8)	1.6 (0.7–3.6)

Bold values indicate statistically significant estimates

Adjusted ORs control for: patient and caregiver age and sex, caregiver education level, patient illness severity (APACHE-II score), reason for admission, and receipt of analgosedative medication

*Abbreviations*: *OR* odds ratio, *95%CI* 95% confidence interval

^a^No delirium was used as the reference category

There was a significant positive association between Sour Seven score and the severity of depression symptoms in family caregivers in the unadjusted model (linear regression coefficient 0.2, 95%CI 0.0–0.4), but the association was attenuated after adjusting for covariates (adjusted linear regression coefficient 0.2, 95%CI -0.0–0.3). There was a significant positive association between the Sour Seven score and the severity of anxiety symptoms in family caregivers both in unadjusted (linear regression coefficient 0.2, 95%CI 0.0–0.4) and adjusted models (adjusted linear regression coefficient 0.2, 95%CI 0.1–0.4). As Sour Seven score increased by one point on the Sour Seven, caregiver anxiety (GAD-7) symptoms increased by 0.2 points.

### Subgroup analysis

For the first subgroup analysis, each feature of the Sour Seven was evaluated individually. After adjusting for covariates, family caregivers who detected* altered level of awareness* (aOR 0.3, 95%CI 0.1–0.9) and *fluctuation* (aOR 0.2, 95%CI 0.1–0.6) had significantly decreased odds of clinically significant symptoms of depression. No other statistically significant associations with depression were observed for the other features of delirium assessed. There were no statistically significant differences in odds of anxiety for any delirium features assessed (Table 4).

**Table 4:** Summary of associations between Sour Seven delirium features and presence of clinically significant depression and anxiety symptoms

	Family caregiver depression		Family caregiver anxiety	
Patient Sour Seven delirium feature	Crude OR (95%CI)	Adjusted OR (95%CI)	Crude OR (95%CI)	Adjusted OR (95%CI)
**1 (Altered level of awareness)**	**0.3 (0.1–0.7)**	**0.3 (0.1–0.9)**	**0.5 (0.2–0.9)**	0.5 (0.2–1.1)
**2 (Reduced attentiveness)**	0.7 (0.3–1.4)	0.7 (0.3–1.6)	0.9 (0.4–1.8)	0.8 (0.4–1.8)
**3 (Fluctuation)**	**0.2 (0.1–0.7)**	**0.2 (0.1–0.6)**	0.5 (0.2–1.1)	0.4 (0.2–1.0)
**4 (Disordered thinking)**	0.6 (0.3–1.2)	0.6 (0.3–1.3)	0.8 (0.4–1.6)	0.7 (0.3–1.5)
**5 (Disorganized behavior)**	**0.4 (0.2–0.9)**	0.5 (0.2–1.0)	0.7 (0.3–1.4)	0.7 (0.3–1.4)
**6 (Impaired eating/drinking)**	**0.4 (0.2–0.9)**	0.5 (0.2–1.1)	0.7 (0.3–1.4)	0.6 (0.3–1.2)
**7 (Difficulty in mobility)**	1.4 (0.5–4.3)	1.3 (0.4–4.2)	0.6 (0.2–1.9)	0.8 (0.2–2.7)

Bold values indicate statistically significant estimates

Adjusted ORs control for: patient and caregiver age and sex, caregiver education level, patient illness severity (APACHE-II score), reason for admission, and receipt of analgosedative medication

*Abbreviations*: *OR* odds ratio, *95%CI* 95% confidence interval

## Discussion

We found that delirium detected by family caregivers using the Sour Seven was not significantly associated with the presence of clinically significant symptoms of depression or anxiety in family caregivers. There was no consistent dose-response relationship between caregiver-detected delirium score and odds of clinically significant depression or anxiety symptoms in family caregivers. When treated as a continuous scale, caregiver-detected delirium score had a significant positive association with depression and anxiety symptom severity.

Family caregivers of critically ill patients experience a high burden of adverse psychological outcomes during an ICU stay, including depression, anxiety, and post-traumatic stress disorder [24]. Our study adds to existing evidence that family caregivers of critically ill patients experience a high burden of depression and anxiety. It is important for clinicians to recognize this psychological burden on family caregivers in order to employ strategies to reduce these burdens, such as providing psychological supports [25, 26] and social work referrals [27, 28]. Further, researchers and policymakers must evaluate and implement strategies to reduce psychological burden in family caregivers of critically ill patients. A recent systematic review and meta-analysis summarized over 100 studies that reported mental health interventions to reduce negative psychological outcomes in family caregivers of critically ill patients [29]. The pooled meta-analysis demonstrated family caregivers experienced reduced symptoms within 3 months after mental health intervention [29].

Numerous studies suggest that delirium is distressing to family caregivers [10–16]. However, few studies have evaluated the relationship between delirium and adverse psychological outcomes in family caregivers. A study in palliative care by Buss and colleagues reported that family caregivers of patients with advanced cancer who witnessed delirium were 12 times more likely to have anxiety compared to caregivers who did not witness delirium [30]. However, this study did not use a validated instrument intended to measure delirium and was performed in a palliative care setting, which may not be generalizable to ICU populations. Our study found associations between caregiver-detected delirium and family caregivers’ symptoms of depression and anxiety. Though, our study was cross-sectional in design and therefore could not determine if delirium preceded depression and anxiety symptoms due to the possibility of pre-existing depression and anxiety symptoms. Additionally, the relatively small sample size of our study may have led to insufficient power to detect significant associations and wide confidence intervals that led to imprecision. For these reasons, further studies using study designs that account for temporality (e.g., cohort study or randomized controlled trial) are required to determine whether family caregivers’ detection of delirium impacts development of depression and anxiety during an ICU stay.

A large body of evidence supports family involvement in patient care to improve patient and family caregiver outcomes [9, 31]. However, it is undetermined whether involvement of family caregivers in detection of delirium symptoms improves family caregiver psychological outcomes through providing a meaningful role in care, or harms family caregivers by drawing attention to distressing symptoms in their loved ones. Family caregivers are motivated and well-positioned to detect delirium in their loved ones due to their familiarity with the patient’s baseline cognition and frequent presence at the bedside [32, 33]. Existing research has highlighted that involving family caregivers in delirium detection using the Sour Seven is feasible and acceptable [7, 34]. The current study highlights the need for a randomized controlled trial evaluating caregiver involvement in delirium detection to evaluate the possible psychological benefits and harms of involving family caregivers in delirium detection.

### Strengths and limitations

This study has several strengths. First, this study was a planned sub-study and analysis of a pre-registered study [7]. Second, this study used validated tools to measure delirium, depression, and anxiety. These tools (the PHQ-9, GAD-7, and Sour Seven) have demonstrated adequate sensitivity and specificity in previous studies, minimizing the risk of misclassification of exposure status (delirium) and outcome status (depression and anxiety), though this is still a possibility with imperfect sensitivity and specificity. Third, this study was conducted in a large academic center with a catchment area of 1.8 million individuals, which led to inclusion of a diverse critically ill population in our sample.

This study has limitations to consider. This study was conducted in a single center, which may limit the generalizability to other settings. This study utilized cross-sectional data, thus we are unable to establish temporality of the association between delirium and depression or anxiety; some family caregivers may have been depressed or anxious prior to the patient’s ICU stay. All questionnaire measures used were self-report, which may lead to misclassification of outcome status (depression and anxiety symptoms). For example, individuals may be more likely to underreport psychological symptoms due to stigma associated with mental health disorders. However, this was likely minimized as participants were given the option to complete questionnaires by themselves and were assured confidentiality of individual information. Although we identified covariates a priori, there may be residual confounding as an inherent risk to observational studies. Lastly, the sample size available for the study resulted in wide 95% confidence intervals. Larger sample sizes should be considered in future studies to increase the precision of effect estimates.

## Conclusions

In this cross-sectional study, we found significant but variable associations between delirium detected by family caregivers using the Sour Seven questionnaire and their own symptoms of depression and anxiety. Further prospective randomized research is needed to delineate associations between patient delirium and adverse psychological symptoms in family caregivers, and to evaluate whether witnessing and measuring delirium may cause adverse psychological outcomes in family caregivers of critically ill patients.

## Data Availability

The data analyzed in the current study are not publicly available due ethical concerns but are available from the corresponding author on reasonable request.

## Abbreviations

ICU: Intensive care unit
GAD-7: Generalized Anxiety Disorder 7
PHQ-9: Patient Health Questionnaire 9
DSM-IV: Diagnostic and Statistical Manual of Mental Disorders, 4th Edition
APACHE-II: Acute Physiology and Chronic Health Evaluation II
